# Evaluation of the salivary biomarker cortisol in patients with temporomandibular disorders

**DOI:** 10.22514/jofph.2026.007

**Published:** 2026-01-12

**Authors:** Bruno Macedo de Sousa, Nansi Lopez-Valverde, Carla Cardoso, Antonio Lopez-Valverde, Maria J. Rodrigues, Jose A. Blanco Rueda

**Affiliations:** ^1^Institute for Occlusion and Orofacial Pain, Faculty of Medicine, University of Coimbra, 3004-504 Coimbra, Portugal; ^2^Department of Surgery, University of Salamanca, 37007 Salamanca, Spain; ^3^Biomedical Research Intitute of Salamanca (IBSAL), 37007 Salamanca, Spain

**Keywords:** Temporomandibular disorders, Stress, Anxiety, Biomarker, Cortisol

## Abstract

**Background**: Temporomandibular Disorders (TMD) are musculoskeletal and neuromuscular conditions involving the temporomandibular joint (TMJ), masticatory muscles, and related structures. Stress can trigger dysregulation of the hypothalamic-pituitary-adrenal (HPA) axis, leading to increased cortisol secretion. Salivary cortisol assessment provides a non-invasive method to investigate this relationship. **Methods**: A total of 98 participants were recruited—49 patients diagnosed with TMD and 49 healthy controls—at the Faculty of Medicine of the University of Coimbra. Participants were evaluated according to the Diagnostic Criteria for Temporomandibular Disorders (DC/TMD). Saliva samples were collected between 9:00 and 11:00 AM, processed with Enzyme-Linked Immunosorbent Assay (ELISA), and analyzed statistically using Shapiro-Wilk and Mann-Whitney tests, with a 95% confidence level. **Results**: Salivary cortisol levels were significantly higher in TMD patients (mean = 17.55 nmol/L) compared with controls (mean = 11.09 nmol/L; *p* = 0.0032). No significant correlations were found between age and cortisol levels. **Conclusions**: Patients with TMD present higher salivary cortisol levels, suggesting dysregulation of the HPA axis associated with stress. These findings support the integration of psychosocial factors into the management of TMD. **Clinical Trial Registration**: ClinicalTrials.gov identifier NCT06874868.

## 1. Introduction

Temporomandibular Disorders (TMD) are defined by the American Academy of Orofacial Pain as musculoskeletal and neuromuscular conditions involving the temporomandibular joint (TMJ), the masticatory muscles, and associated structures of the stomatognathic system [1]. They are the leading cause of non-odontogenic chronic orofacial pain, with a prevalence of 31% in adults and 11% in children and adolescents [2].

The multifactorial etiology of TMD complicates diagnosis and treatment [3]. Early recognition of etiological factors is essential for implementing targeted therapies that can reduce or eliminate symptoms [4]. Risk factors include biomechanical, neuromuscular, psychosocial, and biological contributors. Occlusal changes and parafunctional habits are well-known biomechanical factors [4, 5], while psychosocial influences, such as stress, anxiety, and depression, have become increasingly relevant. Elevated estrogen levels have also been identified as potential biological modulators [4, 5, 6].

Within the biopsychosocial model, pain is understood as not only sensory, but also shaped by cognitive, emotional, and behavioral dimensions. The Diagnostic Criteria for Temporomandibular Disorders (DC/TMD), the current reference standard for diagnosis, integrates physical criteria (Axis I) and psychosocial assessment (Axis II) [7].

Stress activates the hypothalamic-pituitary-adrenal (HPA) axis, leading to cortisol release. This hormone regulates metabolism, immune suppression, and inflammation [8, 9, 10, 11]. Cortisol, synthesized in the adrenal cortex, circulates in both bound and free forms, the latter detectable in multiple biological fluids due to its ability to diffuse through cell membranes [12].

Salivary cortisol reliably reflects serum levels, given passive diffusion from plasma into salivary glands [13]. Because of its non-invasive collection and suitability for repeated sampling, it is widely used to assess HPA axis activity under real-world conditions [14, 15].

A bidirectional relationship between saliva and TMD has also been reported, as alterations in salivary flow or composition may increase joint friction, and inflammatory mediators in saliva can modulate the inflammatory response linked to TMD symptoms [16, 17, 18].

The aim of this study was to compare salivary cortisol levels in patients with and without TMD, to determine whether this biomarker of stress is elevated in affected individuals, suggesting possible hyperactivity of the HPA axis.

## 2. Material and methods

This study was conducted between September 2024 and May 2025 at the Faculty of Medicine of the University of Coimbra (Portugal).

The research project was submitted to the Ethics Committee of the Faculty of Medicine of the University of Coimbra and received approval on 08 April 2024 (CE-059/2024). The study was registered on the platform ClinicalTrials.gov under number NCT06874868.

Participants were recruited from clinical consultations at the Integrated Clinical Unit of the Integrated Master’s in Dental Medicine of the Faculty of Medicine of the University of Coimbra.

Inclusion criteria for the study group consisted of individuals with a confirmed diagnosis of any kind of Temporomandibular Disorder according to the DC/TMD.

Exclusion criteria included individuals under the age of 18, pregnant women, non-autonomous individuals, and those without a confirmed diagnosis of TMD.

The control group excluded individuals with a diagnosis of TMD, complaints of orofacial pain, pregnant women, individuals under 18 years of age and non-autonomous individuals.

The sample size was determined by the availability of patients who fulfilled the inclusion and exclusion criteria during the recruitment period and therefore represents a convenience sample. Although no a priori power analysis was performed, the final sample of 98 participants is comparable to or larger than those reported in previous studies investigating salivary cortisol in TMD. Moreover, the achieved sample size proved sufficient to detect statistically significant group differences.

All participants received a detailed explanation about TMD and the objectives of the study. Those who volunteered, signed an informed consent form freely and knowingly.

Participants were evaluated using the DC/TMD to be assigned to either the study group (patients with TMD) or the control group (individuals without dysfunction). Next, a saliva sample was collected following instructions that included a mandatory 30-minute interval after food or drink intake, and collection was always performed between 9:00 and 11:00 AM to respect the circadian levels of cortisol.

Saliva samples were collected using Salivette® Cortisol devices (Sarstedt, Nümbrecht, NRW, Germany; Order no. 51.1534). The procedure involved placing a swab in the participant’s mouth, who gently chewed it for approximately one minute or until they could no longer resist swallowing. The swab was then removed and placed in the tube, which was sealed and immediately stored at −20 °C until analysis.

To ensure participant anonymity, each individual was assigned an alphanumeric code. Samples were sent for laboratory analysis. Additionally, gender and age data were collected and recorded in Excel spreadsheets for statistical analysis.

This study did not present any risks or potential benefits to the participants.

### 2.1 Instruments and laboratory analysis

The quantification of salivary cortisol was performed using the Enzyme-Linked Immunosorbent Assay (ELISA) technique, recognized for its high sensitivity, specificity, and reliability. This method is based on antigen-antibody reactions, allowing for the quantitative detection of compounds at very low concentrations, such as cortisol.

The kit used for sample processing consisted of microplate strips with eight detachable wells, coated with specific anti-cortisol antibodies. The assay follows the principle of competition, in which the cortisol present in the participant’s saliva sample competes with a known quantity of cortisol conjugated to the enzyme peroxidase for the available binding sites on the immobilized antibodies. After the sample and labeled hormone are added, the plate is incubated at a controlled temperature, allowing effective competition to occur.

After incubation, a careful washing step is performed to remove unbound material, ensuring that only the immune complexes remain attached. Then, a chromogenic substrate specific to the peroxidase enzyme is added. The interaction between the enzyme and the substrate generates a color reaction that is inversely proportional to the cortisol concentration in the sample: the higher the concentration of endogenous cortisol, the less labeled cortisol binds to the antibodies, and therefore the lower the intensity of the developed color.

Absorbance is measured using a spectrophotometric reader at 450 nm, and the values obtained are compared to a previously established standard curve with known cortisol concentrations. This curve allows for the interpolation of sample values, enabling precise quantification of salivary cortisol.

Salivary cortisol concentrations were determined using a commercial ELISA kit (EL2023-0457, DRG® Cortisol Saliva ELISA, DRG Instruments GmbH, Marburg, HE, Germany; Ref. EQ 6141-9601 S). According to the manufacturer, intra-assay coefficients of variation ranged from 3.2% to 4.2%, and inter-assay coefficients of variation from 4.7% to 9.7%, indicating acceptable assay reliability.

### 2.2 Statistical analysis

Descriptive and inferential statistics were conducted with a 95% confidence level (CI). The Shapiro-Wilk test was applied to assess the normality of salivary cortisol distributions. As data did not follow a normal distribution (*p* < 0.05), the non-parametric Mann-Whitney test was used to compare the groups. Effect sizes were calculated using Cohen’s *d*, Hedges’ *g*, and Glass’s Δ. The common language effect size was also reported to facilitate interpretation of group differences.

Correlation analyses between age and salivary cortisol levels were performed using Spearman’s rank correlation coefficient (ρ). Statistical significance was set at *p * < 0.05.

## 3. Results

The study sample consisted of 98 participants, with 49 in the control group and 49 in the experimental group (patients diagnosed with TMD).

In the experimental group, 45 participants (91.8%) were female and 4 (8.2%) were male. The average age was 40.7 years.

In the control group, 44 participants (89.8%) were female and 5 (10.2%) were male, with an average age of 44.2 years.

The demographic characteristics of the sample are summarized in Table 1, presented below.

**Table 1.** Demographic characteristics of each group.

Group	n	Female n (%)	Male n (%)	Mean age (yr)
Study group (TMD)	49	45 (91.8%)	4 (8.2%)	40.7
Control	49	44 (89.8%)	5 (10.2%)	44.2

*TMD: Temporomandibular Disorders.*

### 3.1 Comparison of salivary cortisol levels between study and control groups

In the study group, salivary cortisol levels ranged from 7 nmol/L to 58 nmol/L, with a mean of 17.55 nmol/L and a median of 13.7 nmol/L. In the control group, levels ranged from 3.7 nmol/L to 34.1 nmol/L, with a mean of 11.09 nmol/L and a median of 10.4 nmol/L.

The descriptive and inferential statistics of salivary cortisol levels are presented in Table 2, shown below.

**Table 2.** Descriptive statistics of salivary cortisol levels (nmol/L) in the control and study groups.

Statistic	Control (n = 49)	Study group (n = 49)
Minimum	3.7	7.00
25th percentile	6.5	8.90
Median	10.4	13.70
75th percentile	13.05	20.90
Maximum	34.1	58.00
Mean	11.09	17.55
Standard deviation	5.86	11.93
Standard error of the mean	0.84	1.71
Lower 95% CI	9.41	14.12
Upper 95% CI	12.77	20.98

*CI: Confidence Interval.*

The Shapiro-Wilk normality test indicated *p * < 0.05, confirming that the data did not follow a normal distribution. Therefore, the non-parametric Mann-Whitney test was applied. The test yielded a *p*-value of 0.0032, indicating a statistically significant difference between the groups.

Between-group differences in salivary cortisol were of medium-to-large magnitude (Cohen’s *d* = 0.69, 95% CI 0.28–1.10; Hedges’ *g* = 0.68). Using the control standard deviation (SD) as the denominator, Glass’s Δ was 1.10. The common language effect size indicated a ~69% probability that a randomly selected TMD patient would have higher salivary cortisol than a control.

The distribution of cortisol levels between groups is illustrated in Fig. 1.

**Fig. 1.** Boxplot graph (Cortisol/Control *vs*. Study).

### 3.2 Analysis of the relationship between gender and salivary cortisol levels

The gender-based analysis was underpowered due to the highly unbalanced distribution between female and male participants, and therefore meaningful comparisons could not be performed.

### 3.3 Analysis of the relationship between age and salivary cortisol levels

Spearman’s correlation analysis showed no significant association between age and salivary cortisol levels in any of the groups. In the total sample, the correlation was weak and not statistically significant (ρ = −0.19, *p* = 0.065). Similar results were found in the study group (ρ = −0.15, *p* = 0.312) and in the control group (ρ = −0.21, *p* = 0.153).

The relationship between cortisol and age in the total sample is shown in Fig. 2.

**Fig. 2.** Correlation between age and salivary cortisol levels in the total sample (Spearman’s ρ = −0.19, *p* = 0.065).

Fig. 3 presents the Correlation between age and salivary cortisol levels in the study group. Each point represents an individual participant’s cortisol concentration and corresponding age. The analysis was performed using Spearman’s rank correlation coefficient, which revealed a weak, non-significant negative association between age and salivary cortisol levels (ρ = −0.15, *p* = 0.312). This suggests that, within this sample, age did not have a statistically relevant influence on salivary cortisol concentrations.

**Fig. 3.** Correlation between age and salivary cortisol levels in the study group (Spearman’s ρ = −0.15, *p* = 0.312).

The Fig. 4 shows the correlation between age and salivary cortisol levels in the control group. Each point represents the salivary cortisol concentration and corresponding age of an individual participant. The relationship between these variables was assessed using Spearman’s rank correlation coefficient, which showed a weak negative, non-significant association (ρ = −0.21, *p* = 0.153). This finding also indicates that, within the control group, age was not significantly related to variations in salivary cortisol levels.

**Fig. 4.** Correlation between age and salivary cortisol levels in the control group (Spearman’s ρ = −0.21, *p* = 0.153).

## 4. Discussion

Considering that the diagnosis of TMD, using the most commonly adopted tool—the DC/TMD—must include both physical aspects (Axis I) and psychological factors (Axis II). We believe that evaluating salivary cortisol levels may serve as a useful complementary approach to validated psychosocial questionnaires, providing an objective biomarker that can help to explore the psychological contribution to TMD etiology [17, 18].

In clinical situations where TMD is present without clearly established physical etiologies, salivary cortisol analysis may serve as an auxiliary resource for investigating underlying psychological factors such as stress.

Recent studies have promoted the use of biological biomarkers as diagnostic tools in various joint disorders. In this context, saliva has emerged as a promising biological fluid. The most frequently studied salivary biomarkers include cortisol, alpha-amylase, interleukin-1 (IL-1), and glutamate [19].

Although previous studies have been conducted, there is still insufficient robust and conclusive scientific evidence establishing a clear association between salivary cortisol levels and TMD presence. For instance, a recent systematic review analyzing 14 studies found higher salivary cortisol levels in TMD patients than in healthy individuals. However, the authors highlighted the need for higher-quality, less heterogeneous studies to confirm this association [20].

Our study contributes further evidence supporting the statistically significant association (*p * < 0.001) between TMD diagnosis and higher salivary cortisol levels compared with healthy individuals.

Scientific literature has increasingly identified psychological factors like stress as potential predispositions to developing and perpetuating TMD. The Orofacial Pain: Prospective Evaluation and Risk Assessment (OPPERA) consortium, through a prospective study with 2737 participants over nearly three years, identified stress as a significant predictive factor for developing TMD, along with other pre-existing psychological traits, such as global somatic symptoms [21].

Similarly, Venkatesh *et al*. [22] conducted a study with approximately 350 healthcare students, identifying stress as a relevant etiopathogenic factor in TMD, with muscle disorders being the most commonly observed.

A more recent study assessed the viability of cortisol as a differential biomarker for various TMD subtypes. It found significantly higher cortisol levels in patients with disc displacement without reduction and limited mouth opening, suggesting greater HPA axis activation and dysregulation. Furthermore, it revealed that men with TMD had higher cortisol levels than women, indicating the influence of biological and gender-related factors on stress response [23].

Another study by Chinthakanan *et al*. [24] showed differences in cortisol levels between patients with TMD and healthy individuals, along with significantly higher levels of pain and psychological distress in the dysfunctional group. These results support the hypothesis that cortisol hypersecretion in the early morning may reflect an altered physiological pattern associated with chronic stress and persistent pain.

A recent systematic review, including 11 articles from major databases, corroborates the findings presented here, indicating that TMD patients show elevated cortisol levels compared with those without this condition. The review also reported elevations in other biomarkers, such as interleukin-1, suggesting the concurrent involvement of inflammatory and stress processes in the pathogenesis of TMD [19].

While previous works have synthesized substantial evidence regarding the association between TMD and salivary cortisol, the present study contributes by examining this relationship within a well-defined clinical sample using standardized diagnostic criteria (DC/TMD) and a carefully controlled methodology. This adds value by providing data specific to our population and by reinforcing the importance of integrating biological and psychosocial perspectives in TMD research. Importantly, salivary cortisol should not be regarded as a specific indicator of HPA axis dysfunction. Rather, it represents an accessible biomarker that reflects neuroendocrine activity influenced by multiple physiological and pathological conditions. Our findings therefore suggest that elevated salivary cortisol may signal increased stress-related activation in patients with TMD, although further studies are needed to disentangle its specificity and clinical utility.

Moreover, monitoring cortisol levels during therapeutic follow-up may serve as an objective indicator to adjust treatment approaches, including psychological interventions like cognitive-behavioral therapy and relaxation strategies. Integrating physical and emotional dimensions could significantly enhance treatment effectiveness.

It is also noteworthy that the DC/TMD Axis III is under development, aiming to identify clinically relevant biomarkers, such as quantitative sensory measures and genomic or molecular profiles. This axis seeks to incorporate objective biological information that can assist in diagnosis and treatment, providing concrete data on the patient’s physiological and genetic characteristics. In the future, validated objective biomarkers (Axis III) will enhance the physical diagnostic process beyond current signs and symptoms, leading to more precise understanding and personalized approaches [25, 26].

The finding of elevated salivary cortisol levels in the TMD group compared with controls is consistent with the hypothesis of HPA axis hyperactivity in patients experiencing chronic pain and stress-related conditions. TMD is strongly influenced by psychosocial factors, such as stress, anxiety, and depression, which are known to activate the HPA axis and increase circulating cortisol. This may explain why patients with TMD exhibited higher salivary cortisol values, as chronic psychological distress can act as a sustaining factor in the pathophysiology of these disorders [27, 28].

The absence of a significant correlation between age and cortisol levels may be related to the predominant impact of psychosocial and biological stressors over chronological aging in this sample. In addition, cortisol secretion follows a circadian rhythm and is influenced by a range of lifestyle and behavioral factors, such as sleep quality, diet, physical activity, and medication use, which may outweigh the effect of age alone. These considerations highlight the importance of evaluating psychosocial and behavioral dimensions in parallel with biological markers when investigating the stress-pain relationship in TMD [27, 29, 30].

## 5. Study limitations

This study presents some limitations that should be acknowledged. The sample was of convenience and relatively small, which may affect the generalizability of the results.

Beyond psychological factors, previous research has highlighted additional variables that may affect cortisol levels, including dietary habits (*e.g.*, carbohydrate and caffeine intake), recent physical activity, and the use of medications, such as corticosteroids or psychotropic agents. These potential confounders were not controlled for in the present study [31, 32, 33, 34, 35].

According to the DC/TMD, there are numerous subtypes of TMD that affect men and women differently and present varying prevalences across age groups. In the present study, we did not distinguish between TMD subtypes; instead, they were grouped together into a single category, given the psychological impact that all of them exert on patients [36, 37].

Future longitudinal studies with larger and more diverse samples are recommended to confirm and expand these findings.

## 6. Conclusions

Based on the results obtained in this study, it is possible to state that patients diagnosed with temporomandibular disorder exhibit significantly higher levels of salivary cortisol compared with healthy individuals. These findings reinforce the hypothesis of the involvement of psychosocial factors, such as stress, in the pathophysiology of TMD.

Nevertheless, further studies are needed to more deeply explore the correlation between salivary cortisol levels and clinical indicators of depression and anxiety in TMD patients, in order to broaden the understanding of how psychosocial variables influence the clinical progression of this condition. Additionally, longitudinal studies assessing cortisol levels before and after therapeutic interventions, particularly cognitive-behavioral therapy, should be conducted to evaluate treatment effectiveness.
